# Which neurodevelopmental processes continue in humans after birth?

**DOI:** 10.3389/fnins.2024.1434508

**Published:** 2024-09-06

**Authors:** Shawn Fletcher Sorrells

**Affiliations:** Department of Neuroscience, University of Pittsburgh, Pittsburgh, PA, United States

**Keywords:** amygdala, entorhinal cortex, hippocampus, neurogenesis, ventricular subventricular zone, neuron migration, postnatal maturation, childhood development

## Abstract

Once we are born, the number and location of nerve cells in most parts of the brain remain unchanged. These types of structural changes are therefore a significant form of flexibility for the neural circuits where they occur. In humans, the postnatal birth of neurons is limited; however, neurons do continue to migrate into some brain regions throughout infancy and even into adolescence. In human infants, multiple migratory pathways deliver interneurons to destinations across the frontal and temporal lobe cortex. Shorter-range migration of excitatory neurons also appears to continue during adolescence, particularly near the amygdala paralaminar nucleus, a region that follows a delayed trajectory of growth from infancy to adulthood. The significance of the timing for when different brain regions recruit new neurons through these methods is unknown; however, both processes of protracted migration and maturation are prominent in humans. Mechanisms like these that reconfigure neuronal circuits are a substrate for critical periods of plasticity and could contribute to distinctive circuit functionality in human brains.

## Introduction

The human brain must produce and place billions of cells within the span of months during embryonic development. Like other primates, humans are born altricial and need parental care, so it might therefore be unsurprising that some aspects of neurodevelopment extend well beyond the gestational period. From this perspective, the formation of the brain is constrained by logistical limitations: nervous systems of differing size and complexity require the amplification of different quantities of progenitor cells and have differing constraints on the relocation of their daughter cells to their final destinations throughout the brain. Following this logic, the large and complex human brain just takes much longer to build.

Cross-species comparisons, however, suggest the possibility that this continuing development could have aspects that are more than just logistical. There are multiple challenges associated with building large brains such as dramatic increases in the distances between the birth places of cells and the complexity of the routes that newborn cells must follow to travel to their destinations (Paredes et al., 2016b), but these challenges could themselves be a basis for the emergence of new functionality. There may be significant, even beneficial, impacts to cognition that emerge from delaying—necessarily or otherwise—neurodevelopmental processes into postnatal human life. In the face of environmental perturbations encountered in postnatal life, these continuing forms of plasticity could also be sensitive to, or alternatively, provide resilience to neural circuit structure and function.

## High-demand ventricular real estate

There is a finite amount of room for the radial glia (RG) neural progenitor cells to occupy within the ventricular-subventricular zone (V-SVZ) in the embryo. Particularly for large brains, progenitor populations that do not contact the ventricles, and move into deeper layers of the developing brain, help to amplify neuron production. Interestingly, neurons born directly from RG compared with those born from non-ventricular-contacting intermediate progenitors are not evenly distributed throughout the mouse brain (Huilgol et al., 2023). This demonstrates that differential expansion of progenitor populations to build larger brains is likely to alter the birthplace composition of neurons in different brain regions. In humans, the variety of neurons produced by different progenitors and their differential contributions to brain regions is likely expanded, based on the diversity of progenitor cell types being uncovered (Nowakowski et al., 2016, 2017; Eze et al., 2021; Delgado et al., 2022). Neurons produced from these different birthplaces will encounter a variety of different extracellular environments along their route to their final destinations. It will be important to uncover ways that diverse origins impact the generation and distribution of different cell types throughout the brain.

Most neurons are born a long distance from where they will finally settle. Excitatory neurons destined for the cortex migrate radially along RG fibers, and interneurons migrate tangentially in parallel to the cortical surface. In some instances, the neuronal precursor cells themselves are moved so that new neurons are born closer to their targets. One example of this in the embryonic human brain is outer RG which are unanchored from the ventricle and reposition their soma closer to the cortical plate for additional rounds of cell division. This moves the birthplace of neurons closer to their cortical destinations and increases the effective volume of the germinal niche (Hansen et al., 2010; Lui et al., 2011).

In rodents, the adult dentate gyrus (DG) in the hippocampus also has progenitor cells that are close to the final site of integration of the neurons they produce (Seri et al., 2001). During embryonic ages, these progenitors migrate into the hilus (Li et al., 2013; Sugiyama et al., 2013); however, in humans, they do not appear to completely fill the hilus or converge into a subgranular zone (SGZ) niche. Instead, after humans are born, dividing cells are distributed throughout the DG and decline during infancy (Del Bigio, 1999; Knoth et al., 2010; Dennis et al., 2016; Cipriani et al., 2018; Sorrells et al., 2018). During these ages there are still immature neurons present, but these cells are distributed in a patchwork of clusters across the DG and they decline during childhood (Sorrells et al., 2021). This is quite different from infant and juvenile monkeys where the immature neurons occupy a prominent and contiguous layer interspersed with dividing progenitors (Sorrells et al., 2018). These comparisons hint at underlying developmental differences between humans and other species (Snyder, 2019). If humans do not form a niche that supports a population of progenitor cells in the DG, this could help to explain why neurogenesis is not abundant in adults.

Despite this, there is still disagreement over whether neuronal birth in the adult human DG is abundant (Boldrini et al., 2018; Moreno-Jiménez et al., 2019, 2021; Tobin et al., 2019) or rare (Cipriani et al., 2018; Paredes et al., 2018; Sorrells et al., 2018, 2021; Alvarez-Buylla et al., 2022; Arellano et al., 2022; Franjic et al., 2022). The studies claiming that adult neurogenesis is abundant are inconsistent with one another and with what has been learned about this process in other species. In many instances, the examples of putative new neurons have the morphology of glial cells or large, mature neurons (Boldrini et al., 2018; Moreno-Jiménez et al., 2019; Tobin et al., 2019; Zhou et al., 2022). These examples are located in many different places across the DG and also outside the granule cell layer in the hilus or inner molecular layer (Boldrini et al., 2018; Moreno-Jiménez et al., 2019; Tobin et al., 2019; Zhou et al., 2022). In the adult human hippocampus, no investigations have found a coalesced, proliferative SGZ like what is seen in species where neurogenesis has been verified. One interpretation of this is that, in humans, the neural progenitors do not reside in a coalesced niche, but instead are distributed more broadly. If this were the case, many neurons with the distinctive features of migratory neurons including elongated morphology and migratory gene expression should be present; yet these cells are rare and their identity is inconclusive. Should future work detect such migratory neurons, it will be important to identify their putative sites of birth and include developmental comparisons for context. For instance, a more detailed investigation of the developing human hippocampus might reveal embryonic or perinatal ages when a coalesced germinal zone is still present, which would be needed to uncover the sequence of changes leading to a dispersed niche. It is possible that some of the reported cells in adults are indeed rare newly born neurons; however, the most parsimonious explanation in humans is that these cells are rare and the progenitors decrease along a timeline not unlike what is observed in other primates (Kornack and Rakic, 1999; Charvet and Finlay, 2018; Snyder, 2019).

Transcriptomic approaches offer promise toward the goal of deeper assessment of cellular identity. Single nuclei RNA sequencing (Franjic et al., 2022; Zhou et al., 2022) and spatial transcriptomics (Simard et al., 2023; Ramnauth et al., 2024) analyses have been used to analyze the human hippocampus across postnatal ages. In some instances, analysis of these transcriptomic datasets has identified immature neuronal signatures in adults (Zhou et al., 2022; Ramnauth et al., 2024). Supervised machine learning was needed to classify these cells as different from other granule cell neurons, and their abundance was unclear as these approaches cannot precisely quantify cell populations. Overall these investigations found very low levels of proliferation which indicate that neurogenesis is likely low or absent; it is possible that the detection of neurons with immature properties is the result of a protracted timeline for neuron maturation (Kohler et al., 2011). Protracted neuron maturation has been observed within a subpopulation of excitatory neurons in the amygdala (Martí-Mengual et al., 2013; Sorrells et al., 2019; Page et al., 2022; Li et al., 2023; Alderman et al., 2024; Saxon et al., 2024) and cortex (Zhang et al., 2009; La Rosa et al., 2020; Ghibaudi and Bonfanti, 2022; Li et al., 2023) (considered further in sections below). It will be interesting to compare the immature signatures in the human hippocampus with the protracted maturation occurring in these other regions.

Strikingly, humans are not alone in having limits to adult neurogenesis: dolphins, porpoises, and whales which have large brains and long lifespans, also appear to lack adult neurogenesis (Patzke et al., 2015; Parolisi et al., 2017, 2018). Additional comparisons of the hippocampus niche across a range of mammals could thus reveal additional species where this process is not preserved (Charvet and Finlay, 2018; Bonfanti et al., 2023). It will also be important to uncover the forms of plasticity utilized in the adult human DG and identify developmental mechanisms that result in species differences.

As it is not always practical to move the progenitors themselves, what are some possible alternative ways to recruit new neurons? One possibility is to organize large-scale, long-distance migratory routes for the neurons to follow from their birthplace. Alternatively, brain regions could hold neurons in an immature state until the point at which they are needed. Both possibilities have been found to occur in humans, as considered in the next sections.

## What a long-range trip it’s been

The most prominent postnatal migratory route in rodents is the rostral migratory stream (RMS) which connects the birthplace of neurons in the V-SVZ with their destinations in the olfactory bulb (OB). In the human infant V-SVZ, the newborn neurons migrate in a layer immediately adjacent to the RG (Sanai et al., 2011). Across the first year of infancy, the migration of these young neurons to the OB leaves behind a hypocellular gap between the ventricular wall and the rest of the V-SVZ (Sanai et al., 2004, 2011; Coletti et al., 2018). There are still a few scattered migratory neurons detectable in the adult human V-SVZ as well as some cells that have an appearance of adult neural progenitor B1 cells (Sanai et al., 2004, 2011; Wang et al., 2011, 2014; Coletti et al., 2018; Sorrells et al., 2018). In the macaque V-SVZ there are still many migratory neurons born in adults (Kornack and Rakic, 2001; Pencea et al., 2001; Gil-Perotin et al., 2009), whereas in the marmoset this process is more sharply reduced (Akter et al., 2020). Such observations further point to the need to explore different primates more extensively.

As there are many brain regions far closer than the OB, it is reasonable to wonder whether any V-SVZ derived neurons migrate to destinations in the nearby striatum (Kakita and Goldman, 1999). In rodents, there are neurons migrating from the V-SVZ through the nucleus accumbens to the olfactory tubercle and islands of Calleja in juveniles, with a small number still observed in adults (De Marchis et al., 2004). Cells expressing young neuron markers [e.g., doublecortin (Dcx) or beta-III tubulin (Tuj-1)] and co-labeled with thymidine analogs have been observed in the adult striatum of rodents (Dayer et al., 2005), and rabbits (Luzzati et al., 2006, 2007). Together these studies suggested the possibility that a small population of striatal medium spiny neurons (MSNs) might continue to be born postnatally. More recently, the 5HT3A fluorescent reporter mouse was used to track neurons migrating during adolescence to the nucleus accumbens, and using thymidine analog birthdating, a low rate of neuron incorporation was detected in adults (García-González et al., 2021). The extent to which similar or additional migratory routes are detected in humans and other primates will be interesting to explore. In humans and other primates there is currently less evidence for the addition of adult-born MSNs. No adult-born striatal neurons were detected in monkeys (Wang et al., 2014), despite suggestions of adult-born striatal neurons in humans (Ernst et al., 2014). Further investigations will be needed to resolve whether any striatal neurons are born in adult humans and if confirmed, to identify the precursors for these cells, the extent to which the newborn neurons survive, and the types of neurons and circuits they might grow into.

**FIGURE 1:**
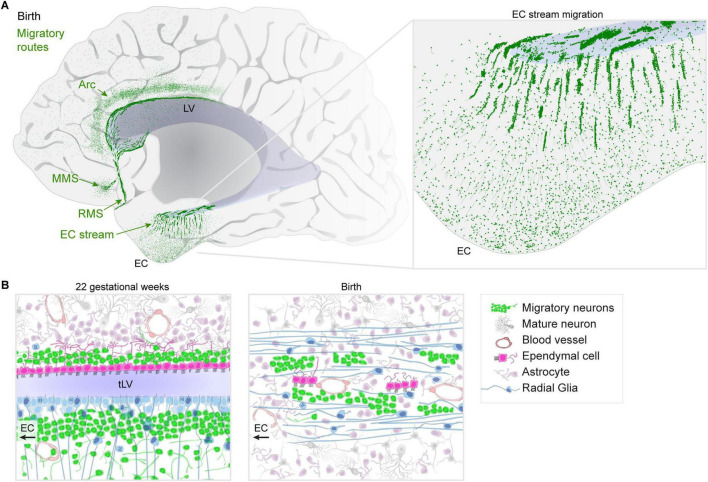
Migratory streams in the human brain at birth. **(A)** Diagram of identified migratory routes present in the anterior human brain at birth. Posterior regions to the right of those shown have been less-comprehensively studied. An “Arc” of neurons extends toward the cingulate and superior frontal gyrus, dorsal to the lateral ventricle (LV). The medial migratory stream (MMS) is directed toward the ventromedial prefrontal cortex (vmPFC) and branches off of the rostral migratory stream (RMS) which delivers interneurons into the olfactory bulb (OB). Inset: the EC stream region shows dense chains and clusters of neurons in a sheet extending away from the temporal lobe lateral ventricle (tLV) medially toward the EC and surrounding regions. **(B)** Diagram of cell types and their organization during mid-gestation when the ventricle lumen in the temporal lobe is open compared to the same region at birth where the EC stream is found. Multiciliated ependymal cells are surrounded by the dense streams of migratory neurons and surrounded by radial fibers from radial glia (RG).

Despite the early postnatal decline in migration to the OB, the human brain has a remarkably diversified array of migratory streams delivering neurons to specific brain regions during infancy. Multiple postnatal streams of migrating neurons have been found in human frontal and temporal lobes (Figure 1A). Just before the decline of the RMS between 8 and 18 months, in 6-month-old infants, a medial branch (the medial migratory stream, or MMS) deviates away from the RMS toward the ventromedial prefrontal cortex (Sanai et al., 2011). This finding prompted a wider search for migratory streams in the frontal lobe which uncovered a large arc of migratory young neurons containing interneurons destined for the cingulate and superior frontal gyrus (Paredes et al., 2016a). Each of these migratory streams is composed of GABAergic interneurons; however, the diversity of neuron types carried by these streams and possible shifts in composition over prenatal and postnatal life are still incompletely understood.

We recently identified a new migratory route in the temporal lobe oriented toward the entorhinal cortex (EC) (Nascimento et al., 2024). Like in the frontal lobe, the majority of these late-migrating neurons are GABAergic interneurons. At birth, the composition of the EC migratory stream is a mixture of cells derived from the medial (MGE) and caudal (CGE) ganglionic eminence germinal zones; however, within weeks after birth, it becomes predominantly CGE-derived. This supports the idea that migratory streams may be traveled by different neurons at distinct developmental stages. Postnatally, the EC stream primarily delivers a type of neuron that expresses the glycoprotein reelin (*RELN*) and lysosomal-associated membrane protein-5 (*LAMP5)* which are prominently found in interneurons in the upper layers of the cortex. What is the difference between how the EC circuits function prior to, and following, the arrival of these cells? How does their arrival alter the properties of EC circuits as they settle and begin to form local connections? Does the conclusion of their integration signal the closure of a critical period for EC plasticity in humans?

Migration through the EC stream extends throughout the first year of infancy, and within the EC itself, some neurons appear to settle until between 2 and 3 years of age. This prolonged arrival may be facilitated by some of the structures that help to establish the route as it forms: during gestation, the ventricle is compressed by the growth of the surrounding tissue. This results in a fusion of two V-SVZs, one from each side of the open ventricle, forming a double-wide collection of dense clusters of migratory neurons (Figure 1B). What are the cellular mechanisms that permit and/or facilitate the fusion of the ventricle in this region, but not others? The V-SVZ is a region where interneurons typically migrate so the ventricular fusion likely paves the highway for migratory neurons to follow; a similar ventricular closure forms the route for the RMS (Sanai et al., 2011). At birth, RG fibers become reoriented toward the EC (Figure 1B), helping to guide neurons away from the ventricle. Some of the multiciliated ependymal cells become subsumed by surrounding tissue, but were not observed in islets like the RMS or MMS. Do these ependymal cells retain any purpose despite their motile cilia becoming embedded within the surrounding tissue? What processes stimulate the young neurons in the dense clusters to disperse once they reach the cortex? Do other parts of the temporal lobe and hippocampal formation receive neurons that migrate through the EC stream?

In some places where postnatal streams are found in the infant human, smaller and more dispersed collections of individually migrating neurons are observed in other mammals including pigs (Morton et al., 2017), rabbits (Ponti et al., 2006), ferrets (Ellis et al., 2019), and mice (Inta et al., 2008). The shared origin of many of the migratory streams in the walls of the ventricles, or formerly open ventricular walls, points to these regions as potentially molecularly permissive to facilitate long-distance, efficient migration, and suggests that they could serve as organizing regions for stream formation. Do major migratory routes arise more frequently in larger brains where tissue growth compresses the ventricle? Interestingly, the rhesus macaque has only a few migrating neurons in the EC stream region at birth. Further comparisons with animal models are needed at embryonic ages to determine whether these migratory routes are simply an extension of embryonic processes, or a novel addition of complexity. In humans, these migratory routes consist of large clusters of neurons (Figure 1A) and the migration extends for months into infancy, making them an exciting target for spatial transcriptomics. Identifying species differences in these postnatal migratory streams or ways they are impacted by the environment or brain injury might further help us understand the significance of the arrival of different cell types at different times into these brain regions (Kim and Paredes, 2021).

The multitude of interneuron migratory pathways extending through infancy raises the question, when and to what extent are excitatory neurons capable of migrating in the postnatal brain?

## Procrastinating neuron development

**FIGURE 2:**
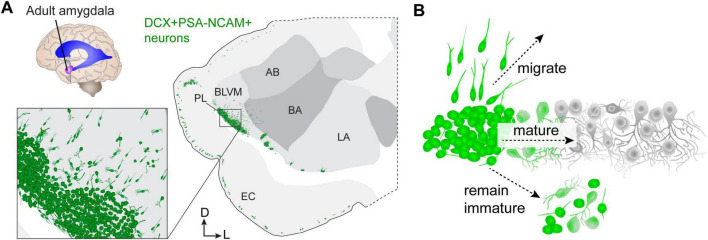
Delayed neuron growth in the human amygdala paralaminar nucleus (PL). **(A)** Diagram of a coronal cross-section of the adult human amygdala showing the location of neurons (green) expressing the immature markers doublecortin (DCX) and polysialylated neural cell adhesion molecule (PSA-NCAM). **(B)** DCX+PSA-NCAM+ cells in the adult human amygdala either continue to migrate into nearby regions, mature locally within the PL, or remain in a simple, immature morphological and molecular configuration. AB, accessory basal nucleus; BA, basal nucleus; BLVM, basolateral ventromedial nucleus; EC, entorhinal cortex; LA, lateral nucleus; PL, paralaminar nucleus.

Some excitatory neurons are also able to reposition themselves in the postnatal brain, however, their migration is generally restricted to nearby regions. An impressive example of this in humans is seen in the amygdala paralaminar nucleus (PL) (Bernier et al., 2002; Fudge, 2004; Zhang et al., 2009; Martí-Mengual et al., 2013; Sorrells et al., 2019; McHale-Matthews et al., 2023) and some regions of cortex in layer 2 (Nacher et al., 2002; Klempin et al., 2011; Rotheneichner et al., 2018; Ghibaudi et al., 2023; Li et al., 2023; Figure 2A). These regions contain neurons that remain molecularly and morphologically immature for weeks, months, or even decades beyond the timeline for when other neurons become mature. But their stasis is not permanent. They begin to increase in size and complexity most rapidly after birth, and more slowly into adolescence, plateauing in adults (Sorrells et al., 2019; Page et al., 2022). Additionally, at every age examined, a subpopulation has the morphology of migrating neurons (Sorrells et al., 2019; Alderman et al., 2024). The presence of so many immature neurons raises questions of whether any are produced from adult neurogenesis (Bernier et al., 2002); however, the majority appear to be born embryonically [reviewed in Page et al. (2022)]. If adult neurogenesis occurs in this region, the neural precursor cells remain to be identified.

Hinting at the importance of delayed maturation as a developmental mechanism, the abundance of neurons undergoing this process is increasingly extensive in the amygdala and is found in more regions of the neocortex in animals with larger and gyrencephalic brains (La Rosa et al., 2020; Ghibaudi and Bonfanti, 2022) including humans (Martí-Mengual et al., 2013; Sorrells et al., 2019) and non-human primates (Chareyron et al., 2012; McHale-Matthews et al., 2023). In mice, similar delayed maturation of excitatory neurons is prominent in the piriform cortex (Rotheneichner et al., 2018; Benedetti et al., 2020; Ghibaudi et al., 2023); however, the mouse also has immature neurons near the amygdala that have homology to those in the human PL (Alderman et al., 2024). Mouse PL neurons exhibit a similar delayed developmental trajectory, express many of the same genes, and have a similar simple morphology compared to those in humans. Unlike humans, the PL in mice contains approximately 10% of the number of neurons as the basolateral amygdala (BLA), whereas in humans it is closer to 20%. This ∼2-fold increase only reflects total neuron number, but there is also an expansion of the location and diffusion of these cells into the amygdala in humans compared to mice. In mice, the migratory neurons emanate into the ventral endopiriform nucleus, which is linked to olfaction, whereas in humans they emanate across the ventro-medial side of the basolateral amygdala (Figure 2A). This difference suggests an expansion of their importance to different types of amygdala processing in humans. Yet, the purpose (Benedetti and Couillard-Despres, 2022) of these neurons being drawn into neural circuits during adolescence is still unknown.

The inference that some PL neurons migrate in juvenile humans is based on the presence of neurons with migratory morphology (Sorrells et al., 2019) as well as increases in mature neuron number with age in nearby amygdala nuclei (Avino et al., 2018). Similarly, in juvenile mice there is a shift in the total number of neurons from the PL into adjacent cortical regions that coincides with the appearance of neurons with migratory morphology (Alderman et al., 2024). This suggests that in both mice and humans, neuronal migration continues in the adolescent brain: a striking form of neuron plasticity. In mice, the route they follow appears as an extension of the lateral cortical migration from the embryo (Bayer et al., 1991; Carney et al., 2006). It has been suggested that this region may be a reservoir for migratory neurons (Bayer et al., 1991); however, if the PL serves as a reservoir, what stimulus is the trigger to halt or re-awaken intracellular migratory machinery?

These observations (Avino et al., 2018; Sorrells et al., 2019; Page et al., 2022) point to three primary fates for the immature PL neurons: either they migrate into nearby regions or they remain within the PL where they either mature or retain an immature phenotype (Figure 2B). The last of these three outcomes is directly apparent: in humans up to 101 years of age (Li et al., 2023), a population of small neurons expressing the immature neuron marker Dcx are still present. Direct evidence of local maturation in mice has been observed in the piriform cortex (Rotheneichner et al., 2018) and in the PL, where neurons can be observed in progressive stages of intrinsic electrophysiological maturation (Alderman et al., 2024). It is also possible that developmental programmed cell death contributes to the decline of immature PL neurons with age (Chareyron et al., 2021). Unexpectedly, however, in mouse PL neurons we observed caspase-dependent programmed cell death primarily at typical ages for cortical excitatory neurons (Alderman et al., 2024). These observations raise additional questions about which stages of development are delayed in these neurons: is their growth of axons and dendrites, or synaptogenesis and pruning also delayed?

## Discussion

After birth, some processes from embryonic development continue in humans. Neurogenesis occurs within the V-SVZ and hippocampus in infants, and sharply drops during childhood to rare or non-existent levels in adults (Sanai et al., 2011; Cipriani et al., 2018; Sorrells et al., 2018, 2021; Snyder, 2019). Migration of interneurons is extended throughout multiple pathways in infants, connecting the germinal regions in the V-SVZ to cortical destinations in the frontal and temporal lobes. Protracted neuron maturation and possibly short-range migration of a subpopulation of excitatory neurons is abundant in the amygdala PL and extends even later into childhood and into adolescence. Differential reliance on these mechanisms in humans could result from constraints imposed by maintenance of neural stem cells: those processes that do not rely on this could be more portable or flexible (Bonfanti et al., 2023). The possible expansion of migratory routes or delayed maturation in species with larger brains, if this continues to be observed across new studies, may be enabled and/or enhanced by the changes that brain growth has imposed.

The prevalence of postnatal migratory streams in human infants raises new questions: are there still more undiscovered regions of the human brain that recruit focal streams of neurons? Do different regions recruit specific subtypes of interneurons? Do other primates or animals have similar (or additional) streams compared to humans? Interestingly, recent transcriptomic and histologic data found that the primate striatum contains subtypes of interneurons that are present in rodents in the OB, suggesting the possibility that these subtypes have been repurposed (Schmitz et al., 2022). This raises the important question of the extent to which postnatal migratory streams might be redirected to alternate regions in different species. Are the final neurons to arrive also the last to be born, or the ones born the furthest away? Is there any difference between otherwise similar neuron subtypes that arrive early compared to those arriving late? The timing of postnatal migration to different regions of the brain hints at its differential importance during critical periods of development. How does the late arrival of these neurons relate to key developmental milestones such as walking or language development? How do environmental influences on these forms of plasticity interact with the emergence of new behaviors?

The expanded presence of the post-mitotic immature neuronal precursor pools in humans, perhaps due to its readily flexible form of cellular plasticity, raises basic questions about its purpose. The migration of PL neurons in humans impacts multiple regions of the amygdala (Avino et al., 2018), whereas in mice migration is largely into the ventral endopiriform cortex. In each case, neurons appear to be flexibly switching their modes of migration as they settle into their final locations, similar to what has been observed for adult-born neurons in the songbird (Scott et al., 2012; Shvedov et al., 2024). The functional importance of the contribution of these neurons to different neural circuits still remains to be worked out, but the rodent and songbird models hold great promise for these goals. The developmental mechanisms directing delayed neuron maturation and the determination of their ultimate fate are similarly unknown. Identifying these mechanisms would reveal basic principles of how the brain develops and could identify new therapeutic targets to promote neuron growth in established brain circuits.

The brain is structurally incomplete at birth, and we have yet to determine the implications of different aspects of development extending into postnatal life. Changes could simply result from finalizing circuit formation, but there could also be as-yet-unidentified special benefits or protection from deleterious influences. Is it an essential or defining feature of human brain development that so many of our brain circuits are completed while we are interacting with our world? In this light, these forms of plasticity could be viewed as an advantage. It could also just be important for neurons to remain structurally flexible when much of their development is continuing postnatally and could be positively or negatively impacted by the world. It is hard to imagine that selective pressure has played no role in these processes because of the timing (childhood through adolescence) and location (memory and emotional brain regions) of these forms of continuing development. Uncovering their significance to humans will provide insights about us all.

## Data Availability

The original contributions presented in this study are included in this article/supplementary material, further inquiries can be directed to the corresponding author.
